# Digital Forensic Analysis of Vehicular Video Sensors: Dashcams as a Case

**DOI:** 10.3390/s23177548

**Published:** 2023-08-31

**Authors:** Yousef-Awwad Daraghmi, Ibrahim Shawahna

**Affiliations:** 1 Computer Systems Engineering Department, Palestine Technical University—Kadoorie, Tulkarem P305, Palestine; 2 Service Delivery Department, ASAL Technologies LLC., Rawabi P666, Palestine; shawahniibrahim@gmail.com

**Keywords:** digital forensics, dashcams, video artifacts

## Abstract

Dashcams are considered video sensors, and the number of dashcams installed in vehicles is increasing. Native dashcam video players can be used to view evidence during investigations, but these players are not accepted in court and cannot be used to extract metadata. Digital forensic tools, such as FTK, Autopsy and Encase, are specifically designed for functions and scripts and do not perform well in extracting metadata. Therefore, this paper proposes a dashcam forensics framework for extracting evidential text including time, date, speed, GPS coordinates and speed units using accurate optical character recognition methods. The framework also transcribes evidential speech related to lane departure and collision warning for enabling automatic analysis. The proposed framework associates the spatial and temporal evidential data with a map, enabling investigators to review the evidence along the vehicle’s trip. The framework was evaluated using real-life videos, and different optical character recognition (OCR) methods and speech-to-text conversion methods were tested. This paper identifies that Tesseract is the most accurate OCR method that can be used to extract text from dashcam videos. Also, the Google speech-to-text API is the most accurate, while Mozilla’s DeepSpeech is more acceptable because it works offline. The framework was compared with other digital forensic tools, such as Belkasoft, and the framework was found to be more effective as it allows automatic analysis of dashcam evidence and generates digital forensic reports associated with a map displaying the evidence along the trip.

## 1. Introduction

Vehicular cyber–physical systems (VCPS) include subsystems of different software and hardware that intelligently cooperate to enhance mobility, safety and entertainment. A dashboard camera (dashcam) is a part of VCPS and is becoming an important in-vehicle accessory for recording audio and visual footage of journeys. In fact, the use of dashcams is increasing rapidly, and the number of dashcams with data, such as GPS coordinates, speed and time, is increasing in the market [1]. Dashcams generate several artifacts of evidential value, such as vehicular speed [2,3], GPS data [4,5], audio [5,6], video [4,5], text data [5,7], objects [8] and static images [4]. Evidence collected from dashcams is more persuasive than other types of evidence, e.g., body camera, due to the absence of perspective bias [9,10]. Therefore, dashcams require the development of applicable, practical and efficient digital forensic solutions [11], and digital forensic investigators have to be aware of the type of the artifact and the format, source and location [4].

The amount of research that is related to dashcam digital forensics is still very small compared with other digital forensic investigation fields, such as computer forensics or network forensics. Dashcam forensics is not included in most digital forensic tools—even commercial ones such as Belkasoft. In most tools, there is no option to select artifacts related to dashcams. For example, when providing a data source (digital evidence) that contains the Windows 7 operating system to a forensic tool, investigators can ask the tool to extract and parse artifacts related to the Windows operating system, such as the registry, user account and browser history. But when the investigator is provided with a data source obtained from a dashcam, e.g., the SD card, the investigator cannot select the artifacts that need to be extracted from the given data source, such as the log files of the dashcam and vehicle trajectories. Thus, the forensic tool will treat the SD card as an attached storage media or data source. Also, experts may use native dashcam video players to view the evidence during investigations, but these players are not accepted in court and cannot be used to extract metadata [4]. Digital forensic tools, such as FTK, Autopsy and Encase, are specifically designed for functions and scripts and do not perform well in extracting metadata, e.g., GPS and license plate data, from a video or images [4]. Therefore, refs. [4,5] show that there is a need for methods that can extract metadata from dashcams. They show that research is needed to identify optical character recognition (OCR) methods that are sufficient for dashcam forensics. Also, dashcam microphones have to be utilized to complete the evidence story, and speech-to-text methods are needed instead of reviewing audio files manually. Further, dashcam forensics methods have to provide the relationship between temporal and geospatial data by utilizing GPS data for map tracking of vehicle locations before and after an incident.

This paper proposes a digital forensics framework that enables investigators to extract metadata related to a specific artifact from dashcam SD cards. Evidential data in dashcam videos are texts displayed on the dashcam screen, and this text represents data that are originally textual, such as the date, time, speed unit and license plate number, or represent vehicle movement, such as the speed and GPS coordinates. Also, evidential data can be warning sounds played by the dashcam about collisions and lane departures or voices recorded by the dashcam microphone. The framework uses OCR to extract time, date, speed and GPS coordinates from the watermark information. The framework also converts the speech played for lane departure and collision warnings and the speech recorded by the microphone into text. The text can be easily indexed and searched and is used to generate the digital forensic reports that are accepted in the court. The framework associates the metadata to a map that allows investigators to track evidence on the entire trip. For each investigation session, the framework displays a forensic report related to the investigated video files.

The proposed framework was evaluated by selecting a dashcam for experiments. Three OCR methods—[12,13], GOCR [14] and OCRAD [15]—were tested and compared. Tesseract was found the most accurate method for extracting evidential text from dashcam videos. Also, two speech-to-text methods—the Google speech-to-text API [16,17] and Mozilla’s DeepSpeech [18]—were tested. The Google API was found to be more accurate, while DeepSpeech is more acceptable by investigators because it works offline. When comparing the results of the proposed framework with other digital forensic tools, the proposed framework outperforms the others as it understands the given video file from the storage media and extracts artifacts specific to the dashcam model. The overall flow of the investigation framework follows the NIST 800-86 recommendations published in [19].

This paper contributes to the digital forensic research field and the technical field. Since few resources discussing dashcam forensics can be found in the literature, the paper is considered a source of knowledge about the use of dashcam digital forensics and the type and the source of artifacts and how to acquire them. The paper compares different OCR methods and speech transcription methods and identifies the best for dashcam forensic analysis. The paper is the first in this field that presents automatic dashcam voice transcription for avoiding manual analysis, which is difficult and time-consuming. Technically, this paper proposes a forensics framework that extracts evidential text and speech metadata from recorded dashcam videos in a short time. In fact, the proposed framework is a new methodology that enables collecting all metadata related to one evidence source in one place instead of using different software tools. This also ensures the authenticity of evidence by reducing the chance of modifying artifacts via different tools.

The rest of this paper is organized as follows: Section 2 presents the related work. Section 3 explains the proposed dashcam investigation framework. The experiments are described in Section 4. Section 5 presents the results, and finally, Section 6 concludes the research.

## 2. Related Work

This section presents a brief history and the importance of dashcams. Then, the section shows the most-related research in the field.

### 2.1. Dashcam Technology and Forensic Analysis

Video sensor digital forensics have become necessary since the start of closed-circuit television (CCTV) cameras. With the emergence of CCTV, crime detection became less time-consuming, as CCTVs have been installed everywhere from homes to public places [20,21]. CCTVs have been considered a great strategy for analytical improvement in crime detection, as they enable temporal and spatial analysis [22,23,24]. The United Kingdom CCTV industry showed that with the installation of CCTV systems, the crime rate decreased significantly [25,26]. CCTVs require video processing techniques for the analysis and identification of crimes [27].

In the vehicular context, different sensors are crucial sources of digital evidence [28]. One of these sensors is the dashcam, which has been used for multiple purposes [29,30,31,32]. Dashboard cameras have been used to increase road safety [33]. These cameras are able to record both audio and video and are mounted inside vehicles. Most of the cameras are facing forward, but some of them are dual-lens cameras that can provide footage of what is happening in front of and behind the vehicle while driving. Dashboard cameras are either mounted on the windshields or in front of the vehicle [34].

Nowadays, dashboard cameras are integrated with built-In GPS, which can provide speed, time, location and route data [35]. The dashcam industry is evolving rapidly, as the number of vehicles with installed dashcams and the expected market size will reach $5.2 billion by 2026 [36]. Dashcams provide a wide variety of features that are available and are associated with the recording purpose, including, but not limited to, GPS location of the recorded video footage, car speed during video footage, connection to personal assistance like Amazon Alexa, mobile hotspots, voice commands, emergency events, accident detection, parking mode, G-sensing, and auto-sync with the cloud [37,38]. Dashcams can be controlled through the camera itself if the dashcam has a screen, and/or a mobile application can be installed on a mobile device to configure the dashcam.

Dashcams are the most useful resource for vehicular crime events and accidents as the recorded footage not only provides quantitative data of car movement but also provides additional information like the driver’s vision condition [39]. The footage from dashboard cameras is stored in the memory, and this footage can be used by courts for testimony to remove uncertainties and by insurance companies. Video recording from an installed camera in a vehicle helps insurance companies deal more quickly with damages issues [40]. Dashcams gain huge significance as they help forensic experts in reconstructing road crashes and preventing distortion of the facts, which could lead to a possible miscarriage of justice [41,42]. A study shows how a dashcam was proved to be great evidence for putting on trial a ‘crash for cash’ fraudster who intentionally caused a car accident just to claim the insurance money [43]. Also, video evidence can clarify many cost claims by providing important details about the incident: for example, third-party pedestrian claim costs drop by 80% for vehicles with dashcams installed [44].

Ensuring the authenticity of the evidence retrieved from dashcams is important. It needs to be evidence generated by the dashcam itself and not modified by any external party [45]. This requires all available artifacts extracted from a dashcam to be connected together. For example, a fraudster can submit a video recorded by another dashcam, causing the case of a staged accident. When performing a digital investigation for the dashcam installed on the car that the fraudster claims is recorded on the video, other artifacts related to the video need to be consistent. The GPS data should point to the location of the accident, and the logs of the dashcam should indicate an emergency event that happened. When there is inconsistency between the submitted video evidence against other artifacts related to the video recording, this can be considered as fraud.

Few studies have discussed dashcam digital forensic analysis. Ref. [4] described the artifacts extracted from dashcam SD cards related to the recording mode of the camera, the GPS data associated with the recorded videos, data related to the vehicle speed and license plates that were captured by the camera. Although [4] could find a number of artifacts from the SD card of the cameras, the research did not include artifacts from the dashcams themselves or explore the logs written by the dashcams and/or the mobile applications associated with the dashcams. Additionally, refs. [4,5] stated the need for dashcam forensic methods that use OCR to extract metadata, microphones to record surrounding sounds, and associations between temporal and geospatial data.

The authors in [45] proposed an algorithm to extract features from the videos that are uploaded online into a publicly accessible website. The feature extraction relies on recognizing the motion blur in the video recording that is generated by the unique movement of the vehicle while the dashcam is recording. This helps digital forensic investigators identify the source vehicle from which a certain video is recorded in case that video is used as evidence in an insurance claim or as evidence in a court. Further, an open-source framework was proposed to identify a variety of metadata related to 14 types of dashcams associated with a database to store the metadata [46]. Although this study proposed systems that are able to recognize and store a lot of metadata related to different dashcams, it was not able to recognize the dashcam from which the digital evidence is acquired. Also, the authors do not support review of logs, which makes the investigations more difficult. According to [45], digital investigations need an algorithm that helps to prove if the submitted video recording is generated by the same car that had the accident.

Recently, online submission of digital forensic evidence has emerged to enable rapid investigations. This is also applied to dashcam forensics, as witnesses can upload evidence videos to portals through which law enforcement agencies can access these videos. Although online submission of digital evidence facilitates digital investigations, the increase in the number of submissions requires more processing time and memory [4]. Also, online submissions pose serious challenges to investigations, as digital evidence can be modified [47], shared with third parties [48], superannuated and manipulated [49]. Due to these reasons, this research uses offline investigations by direct reading of the dashcam SD cards.

In summary, dashcams are becoming more widespread than before, and they contain important evidence regrading vehicle incidents. Dashcam videos include several artifacts that assist digital forensic investigations. These artifacts require extraction methods that are accurate and that ensure integrity and authenticity. The most important sources of artifacts in dashcam videos are the text that expresses GPS coordinates, speed, time and date, and the speech that expresses warnings about lane departures and collision possibilities.

### 2.2. Dashcam Text and Speech Analysis

Dashcam digital forensic investigation requires extracting text from videos and analyzing speech. In relation to text extraction from video evidence, the authors of [50] developed a cloud-based OCR to extract text that is necessary for digital forensics. This method depends on online processing, which poses security threats to the analysis [49]. Ref. [51] presented the challenges in license plate recognition using OCR. Internal challenges are related to the software used for processing and the camera hardware and include resolution, view angles, focus speed, the processor and internal memory. External challenges are related to plate variations (font, color and plate position), environment factors (lighting conditions and surrounding effects) and camera mounting variations (inclination and distance from the plate). Also, ref. [52] used OCR to extract timestamps from videos to sort them according to time for digital investigations. However, the type of OCR used for video forensic analysis is not specified well in these studies. The research in [53] shows that OCR methods on images requires much computational time, and [54] shows that OCR methods can achieve high accuracy in different weather conditions if they are integrated with deep learning.

In relation to dashcam speech forensic analysis, ref. [5] states that the recorded speech on the dashcam about lane departure warnings and collision warnings is still analyzed manually. There is a need for digital tools that help investigators to analyze the speech and generate the forensic report. In other digital forensic domains, voices and sounds are considered important because they contain relevant evidence for the investigation. So automatic speech recognition that transcribes speech-to-text is necessary for digital investigations because text is predominant in digital investigations and the most acceptable type of evidence in courts [18]. Also, text can be parsed, indexed and searched easily, while integrating voice with digital forensic examinations is difficult [18]. The process of speech conversion to text is composed of speech detection and speech transcription. Although a number of libraries are available for speech detection and transcription, few are offline and open-source and support digital forensics without referring to third-party services in the cloud. Digital forensics require that the speech is not modified. For ensuring audio integrity, the authors in [55] propose a method that uses hash values to verify if audio has been modified or not.

In summary, the aforementioned studies did not sufficiently describe the methods used for text or speech extraction. This research agrees with [4,5], which states that there is a need for identifying the best text recognition methods and speech-to-text conversion methods that can be used to extract forensics metadata from dashcam videos. To address the problems in these studies, this research proposes a solution to investigate the storage media acquired from dashcams. The proposed solution is a dashcam forensics framework that is able to extract artifacts from given dashcam evidence and enable investigators to associate the dashcam and the extracted metadata with a map location that supports evidence tracking. This research examines different text and speech extraction methods, compares them, and identifies the one that can achieve better results than the others.

## 3. Dashcam Investigation Framework

Digital forensics is the branch of forensic science that intends to uncover and investigate the materials located inside digital devices associated with a cybercrime [56]. Digital forensics can be performed in different ways, and the National Institute of Standards and Technology (NIST) recommends a process that goes through four steps for conducting digital forensics, and these steps are [57]:

- Collection—Identifying, acquiring and protecting the data collected at the crime scene;
- Examination—Processing the collected data/evidence and extracting relevant information;
- Analysis—Analyzing the extracted information in order to connect the dots and be able to build a robust and admissible case;
- Reporting—Presenting the findings of the analysis stage in an admissible and understandable format.

This research follows the guidelines published by the NIST 800-86 [19]. This section presents the design of the proposed dashcam forensics framework: i.e., the implementation architecture, including the structure and execution details.

### 3.1. Framework Design

The purpose of designing the investigation framework is to extract metadata associated with dashcam-recorded videos for generating a digital forensic report. The input to the framework is a video that contains evidence incorporating metadata representing the vehicle’s status along the trip. The video contains text data that are originally represented by text on the screen, such as the time, date and license plates of other vehicles. Also, the video contains text representing vehicle movement, such as the speed, speed unit and GPS coordinates (longitude and latitude). The video also contains sound that represents vehicle movement, such as collision warnings, overspeed warnings and lane departure warnings played by the dashcam itself. The sound can also be voices of the driver and passengers recorded by the dashcam microphone. These data should be extracted and processed in the framework to generate an output containing a full digital forensic report that shows the evidence along the trip. Also, because vehicles are moving objects, the framework should be able to identify the temporal and geospatial relationship by associating the recorded video with a map.

The framework should allow investigators to perform a visual review of the recorded videos and then to add the required details that need to be extracted to each video as a stamp. Based on the given information on the stamps, the framework splits each recorded video into frames. The number of frames per second can be adjusted by the user to generate a reasonable amount of interactive points that can be added to the map for identifying vehicle trajectories during the video recording. The default value is set to one frame per second.

#### 3.1.1. Extracting Evidential Text

**Figure 1:**
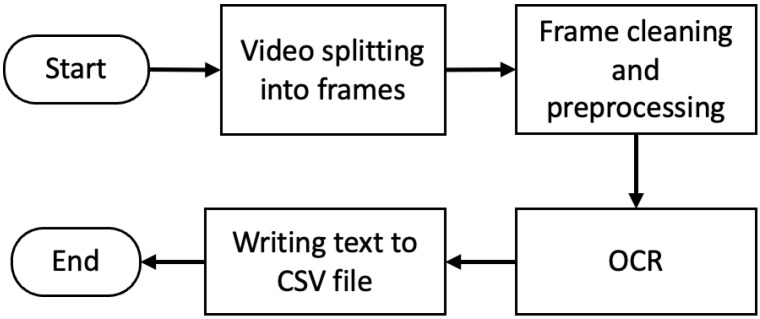
Flowchart of extracting evidential text from a dashcam video.

The extraction process starts by processing the video to extract text metadata from the video watermark via optical character recognition (OCR) techniques. Figure 1 shows the flowchart of the action sequence taken for extracting the desired data from a video clip that has been split into frames. The first step in the proposed framework starts by cleaning each frame to remove noise and pixels that do not contain relevant details. This is a pre-processing stage for noise reduction and image stabilization. We used OpenCV for framing the video and cleaning the frames.

Once the frame cleanup process is done, OCR is used to extract the stamp within the cropped area out of the frame. This research uses different OCR techniques to extract text, including Tesseract [12,13], GOCR [14] and OCRAD [15]. After the text is extracted from the frame, the content of the text is split into *Date*, *Time*, *Longitude*, *Latitude*, *Speed*, *Speed unit* and *License plate*. These parameters are stored in a CSV file. The text extraction process terminates when the text is extracted from the last frame.

**Figure 2:**
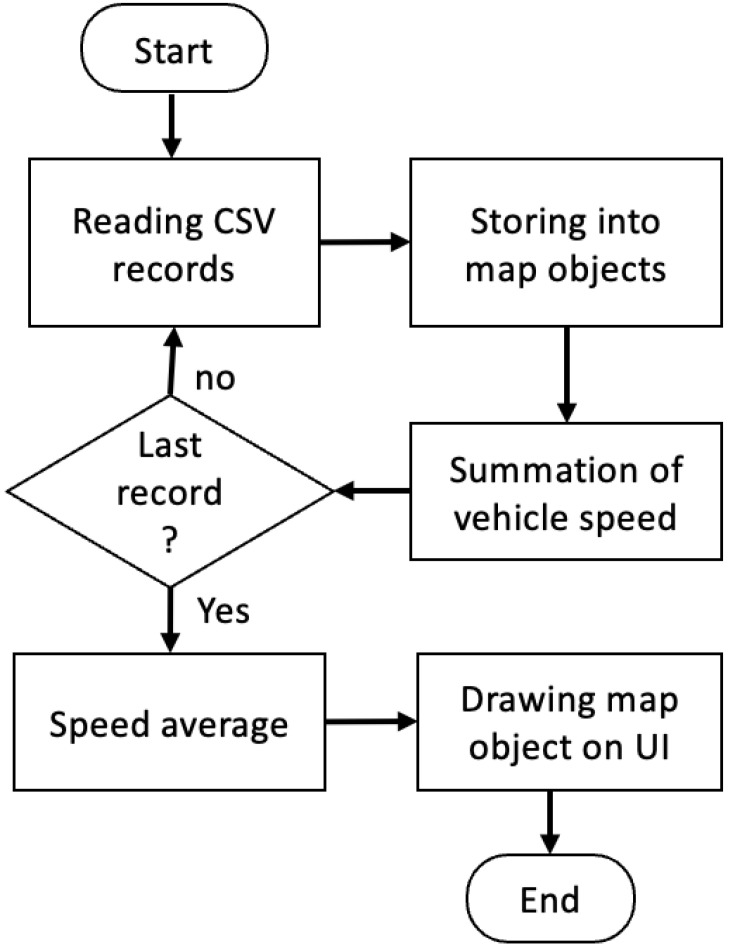
Flowchart of drawing the extracted evidential metadata on the map.

The CSV file generated in Figure 1 contains multiple records, and each record has to be read separately for drawing the vehicle trajectories on a map. Figure 2 shows the flowchart of drawing the extracted coordinates and time stamps onto an interactive map. The algorithm described in Figure 2 loops over the stored data in the CSV file. It draws each coordinate point (longitude and latitude) and the associated data on an interactive OpenStreetMap (OSM) file, and it calculates the average speed of the vehicle during the recorded video as:

(1) $$\begin{array}{c} AverageSpeed=\frac{S1+S2+\ldots +Sn}{n} \end{array}$$

where *S* is the speed of the vehicle in each frame and *n* is the number of frames. Then, the design incorporates adding the extracted information to the map.

#### 3.1.2. Extracting Evidential Speech

The framework allows conversion of video speech into a text file to detect lane departure and collision warnings. The framework transcribes the content of the video to be added to the final forensic report, which is necessary for digital investigators. Also, the dashcam contains vocal warnings that need to be transcribed into a text file for further processing if needed by the court. The other reason for converting the video speech into a text file is to enable translating the notifications and warnings extracted from the dashcam into other languages. The default language is English, which can be translated to other languages easily using any open-source translation library.

**Figure 3:**
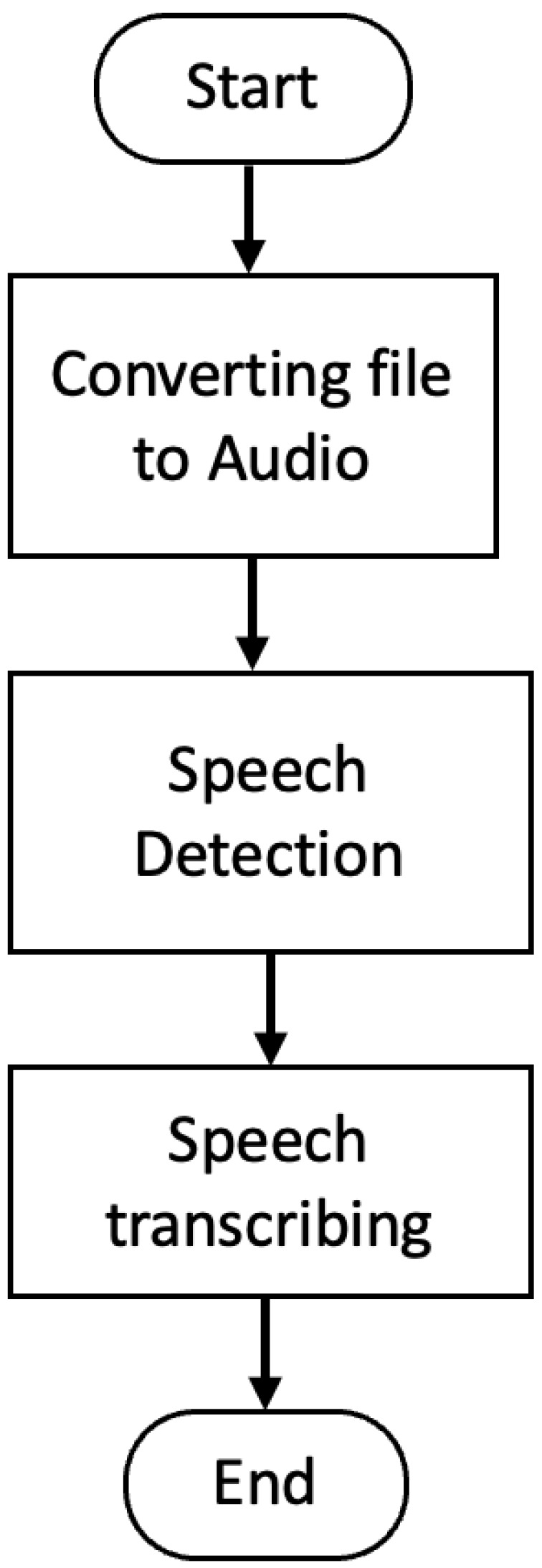
Flowchart of converting the video speech into a text file.

The speech-to-text conversion starts by converting the video into an audio file. The framework then starts recognizing the text from the audio file. For achieving this, we use the open-source speechRecognition software [16,17] that is a wrapper of different APIs, such as the Google speech recognizer API. Also, we use the INA’s inaSpeech Segmenter [18] for speech detection and Mozilla’s DeepSpeech [18] for speech transcription. We compare both the Google API and DeepSpeech. Figure 3 shows the flowchart for converting the speech in a selected video file into a text file based on the selected locale.

**Figure 4:**
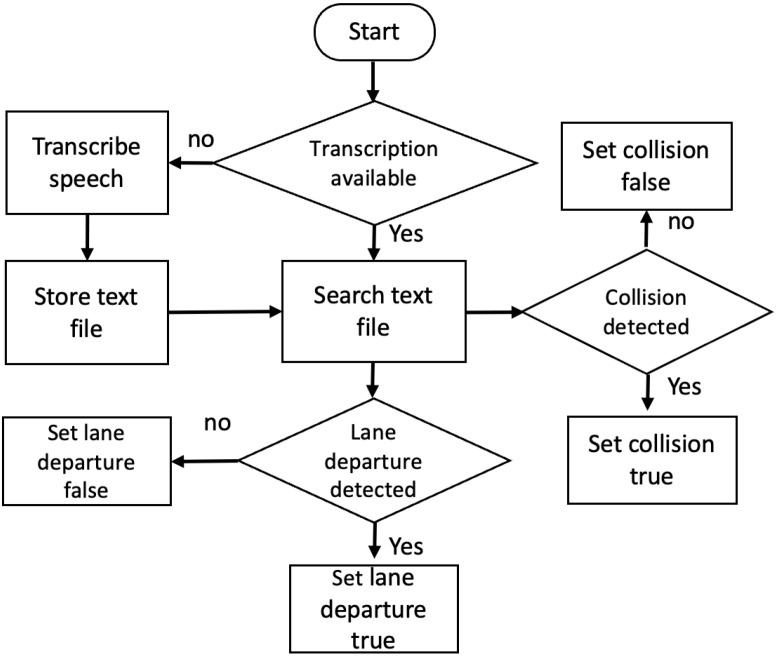
Flowchart of detecting lane departures and vehicle collisions.

After transcribing the video speech, the framework can detect lane departures and collisions by searching for the related key words. Figure 4 shows the flowchart for detecting lane departure and collision warnings and for storing the results into variables to be reflected in the final digital forensic report.

### 3.2. Framework Implementation

The dashcam forensics framework is implemented using Python 3.7 as a programming language to support the available digital forensics libraries. PyQT5 is used to implement and generate the graphical user interface (GUI). The QT designer is used for designing the GUI of the project, which is compiled during the run-time. During the implementation, other Python packages, such as pyewf and pytsk3, are also used to facilitate handling the evidence images under investigation. The purpose of the two packages is to allow Python to interact with the provided evidence images. The implementation uses another Python package that is capable of building PDF files in order to generate the final digital forensic report.

The framework project consists of a set of Python files and directories. The classes of the project are distributed into multiple Python files and directories that are used for storing different outputs during the processing of the artifacts extracted from the provided evidence image. The main directories are listed under the project root directory, and they include:

- audio_files: this includes the audio files stored as an intermediate stage while converting the video to text;
- evidence_utils: this includes two Python classes: ImageMounter is responsible for mounting the E01 image and loading it into the operating system with the assistance of the EWFImgInfo class that is for reading operations on the image;
- exported_coordinates: this stores the CSV file that is generated after extracting the vehicle speed, time, date, longitude and latitude from the selected video;
- exported_videos: this stores each file exported by the framework for processing;
- map_file: this stores the generated preview.html file, which is the interactive map displayed to the investigator via the GUI after processing the selected video;
- out: this stores the frames that are exported from the selected video and are cropped;
- recognized_audios: this stores the text files that are generated by audio-to-text implementation, storing both Arabic and English transcribed files for the video with the following names: arabic_audio_transcript.txt and english_audio_transcript.txt, respectively;
- ui: the design files created using QT Designer for the GUI are stored under the ui directory. The directory includes the following files, which are compiled on run-time: file_explorer_widget.ui, main_screen.ui and partition_table_widget.ui.

The main project files are listed under the project root directory, and they include:

- draw_on_map.py: this reads the generated CSV file that contains the vehicle speed, time, date, longitude and latitude, which are extracted from the selected video file. It also stores them as an OpenStreetMap object and translates the coordinates into the interactive HTML file preview.html to be displayed on the GUI;
- generate_report.py: this uses the Python package FPDF for writing texts and images related to the selected video artifacts into the report object and converts the object into a PDF file when generating the digital forensic report;
- img.jpg: the temporary file is generated when the user clicks the “Add To Report” button in order to add the interactive map snapshot to the report file;
- Investigation_Report.pdf: this is the PDF report that is generated when the user clicks the “Generate Report” button;
- ocr_extractor.py: this set of functions is responsible for framing the selected video file, cropping the frames and extracting artifacts from each frame;
- main.py: the main file of the project that starts the execution and holds the variables that store the artifacts for each selected video and the classes for the GUI interactions;
- speech_recognizer.py: the set of functions responsible for converting the selected video into text by converting it into an audio file using the moviepy Python package and storing it in the audio_files/audio.wav directory. It also contains the speech_recognition Python package to convert the language into an Arabic or English text file based on the selected localization.

## 4. Experiments

This section focuses on setting up the experiments, including the dashcam installation. Then, it discusses the procedure of generating dashcam digital evidence that is used as investigation material for the framework. To perform the investigation, the research builds an investigation machine that is capable of performing the needed evidence acquisition and evidence processing and generating the digital forensic report.

### 4.1. Experiment Settings

For the purpose of evidence acquisition, analysis and reporting, a platform is setup with hardware and software specifications as follows:

- CPU—Intel Core i5 10400f 2.90 GHz;
- RAM—16 GB DIMM 2666 MHz;
- Storage—256 GB M.2 SSD;
- Operating System—Windows 10 Pro Version 1909;
- Acquisition and Analysis Software—Belkasoft Evidence Center, Version 9.9 Build 4662 ×64 [58];
- Acquisition and Analysis Software—Magnet AXIOM, Version 4.6.0.21968 [59];
- Acquisition Software—AccessData FTK IMager, Version 4.5.0.3 [60];
- Investigation Software—Autopsy Digital Forensics, Version 4.16.0 [61];
- Investigation Software—Registry Viewer, Version 1.8.0.5 [62];
- Virtualization Software—VMware Workstation Pro, Version 16.1.0 Build 117198959 (×64) [63].

We select a dashcam running the Android 8.1 operating system, which covers as many of the features that can be generated from a dashcam as possible in order to provide artifact coverage for efficient evidence processing and artifact extraction. The dashcam model is the Bluavido AC29 equipped with an advanced driving assistant system. The dashcam features support for 4G and WiFi connectivity, allowing it to be controlled remotely using a mobile phone.

**Figure 5:**
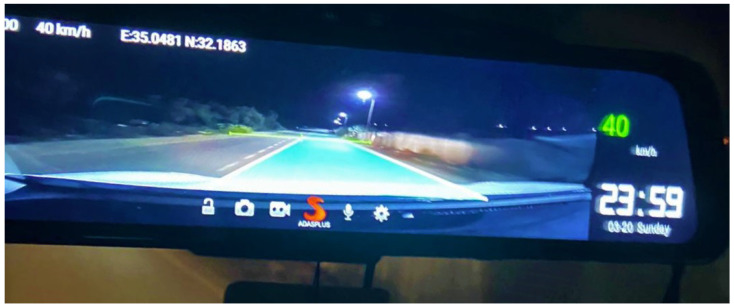
Lane-tracking feature of the dashcam.

The dashcam provides the feasibility of recording the longitude, latitude, traveling speed, speed unit, license plates of other cars, date and time as a digital stamp written over each video produced by the dashcam. At the same time, the dashcam performs real-time processing for the video feed coming from the front camera. The real-time processing allows the dashcam to draw a light blue line that highlights the lane in which the vehicle is traveling, as shown in Figure 5. This feature allows the dashcam to play a vocal notification to the driver saying “Lane Departure” when the driver drifts out of the lane.

Another feature of the real-time processing is collision detection. The dashcam can detect other vehicles on the road, and when the driver gets close to these vehicles, the dashcam highlight them with light-blue circles. If the driver gets closer and closer, the circle highlighting the detected vehicle becomes orange and then red and will show an approximate distance between the vehicle with the dashcam and the detected vehicle. When the dashcam detects vehicles on the street with a certain collision probability based on the measured distance, the dashcam plays a vocal warning to the driver of “Attention to the cars” to indicate a probably collision.

### 4.2. Experiment Process

This research uses a dashcam that outputs one of the most widely used dashcam data types. This enables us to generalize the results in the future. The dashcam comes with connectors that can be connected to the battery of the vehicle with three wires. One goes into the 12 V connector to ensure the camera is always on. Another wire is connected to the ACC to turn on the dashcam when starting the vehicle, and the third wire is connected to the body of the vehicle to complete the circuit. An external 220 V AC to 12 V DC converter is used for the purpose of running the dashcam without relying on the car battery and to ensure the dashcam has no defects.

To ease with dashcam installation and relocation from one vehicle to another, the three wires are connected together in a 12 V cigarette lighter plug. The reverse/rear-view camera is connected to an external switch that simulates the behavior of turning the video source of the dashcam to use the reverse camera when the driver puts the vehicle into the reverse driving gear. The reverse camera is connected to the external button and into the dashcam input slot to feed the rear camera view. A SIM card that supports a 3G mobile data connection and a 16 GB class-10 SD card are installed inside the dashcam inputs. The GPS antenna that comes with the dashcam is installed as well into the dashcam inputs.

To generate experiment data, the dashcam is installed into a vehicle, and all data-generation steps are processed with extreme caution and under controlled conditions. The dashcam is used to record a wide variety of videos at different speeds and road types plus departing a lane to have a data set containing all the features provided by the dashcam. The last step in the experiment is to collect the data from the dashcam for performing the investigation. The SD card is ejected from dashcam slot and is connected to the investigation workstation demonstrated in Section 4.1.

The collection process is performed through the following steps:

- Running write-blocker software on the investigation workstation to prevent SD card alteration once connected to the investigation workstation;
- Connecting the SD card to the investigation workstation using an SD-card-to-USB adapter;
- Opening storage media acquisition software (FTK Imager);
- Creating a forensics image from the SD card using Expert Witness Format (E01).

**Figure 6:**
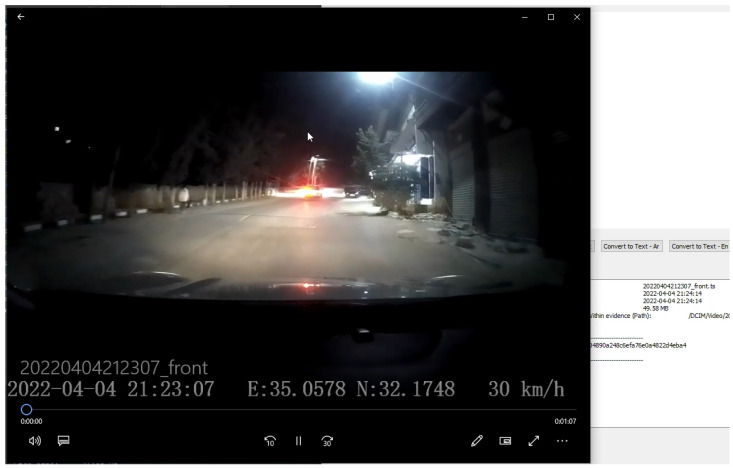
A screenshot from a selected video file played in a native video player.

The framework allows investigators to open the recorded video in a native video player, as shown in Figure 6. The video is the input to the text extraction and speech conversion processes. For text extraction, three methods—Tesseract, GOCR and OCRAD—are run and compared. For comparison purposes, we define character accuracy as the percentage of correctly recognized characters compared to the ground truth for each frame, i.e., for each second, as the default setting is one frame per second. We also compute the average of character accuracy for the entire video as the sum of frame accuracies over the number of frames.

For speech-to-text conversion, we run speechRecognition [16,17]—Google’s speech recognition API. We also implement INA’s inaSpeechSegmenter [18] for speech detection and Mozilla’s DeepSpeech [18] for speech transcription. We compare both the Google API and DeepSpeech. To evaluate the accuracy, we use the Word Error Rate (*WER*) [64], which is calculated by the formula:

(2) $$\begin{array}{c} WER=\frac{lev(t)}{len(r)} \end{array}$$

where *r* is the reference text, *t* is the transcribed text, *len* is the number of words in a sentence, and *lev* is the Levenshtein distance at the word level. A video that contains lane departure and collision warnings and the driver’s voice was used to test the speech-to-text conversion.

## 5. Results

This section presents the results obtained from the research. The results are divided into text extraction, speech conversion and time and memory review.

### 5.1. Text Extraction Results

**Figure 7:**
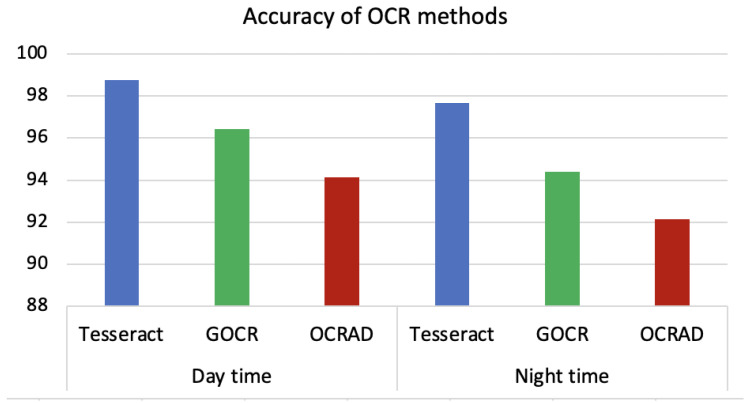
The comparison of OCR methods on videos recorded during day- and nighttime.

The results of using Tesseract, GOCR and OCRAD for extracting forensics metadata from dashcam videos recorded during daytime (left side) and nighttime (right side) are shown in Figure 7. The figure shows the average character accuracy for the entire video. Both videos were recorded at a vehicle speed of 40 km/h. The frame settings were adjusted as color images with brightness = 50 and resolution = 300.

The figure shows that Tesseract outperforms GOCR and OCRAD in both scenarios. GOCR achieves better results than OCRAD. However, the accuracy of all methods decrease in the videos recorded at night. This reduction in accuracy is due to the lighting conditions, which agrees with [51]. Tesseract still achieves high accuracy during day- and nighttime, while the accuracy deterioration of GOCR and OCRAD is greater than that of Tesseract. This makes Tesseract the best candidate to be used for digital dashcam video forensics. Tesseract can extract forensics metadata related to time, date, latitude, longitude, speed and speed unit from the dashcam video.

### 5.2. Speech Transcription Results

The aim of speech conversion is to convert the warning sounds played by the dashcam about lane departure and collision possibility into text. These two variables are very important for digital investigations related to vehicle incidents, and they have to be in the digital forensic report. We calculated the WER for the Google API and Mozilla’s DeepSpeech on different dashcam videos with different lengths. Table 1 shows the WER values of Google API and DeepSpeech for different videos of different lengths.

It is clear that the Google API outperforms DeepSpeech because the WER in all cases is lower. It is worth noting that the video length does not affect the WER because the values in the table are influenced by the number of words in each video. DeepSpeech is still a viable option for dashcam digital forensics, particularly because it is open-source and offline, while the Google API requires cloud services.

### 5.3. Time and Memory Review

This section introduces the performance analysis for the functions used in processing the content of the video, transcribing the video and generating the digital forensic report. The execution time analysis is conducted using the line_profiler Python package that tracks the execution time for each code line and provides an overall execution time for the profiled function then uses an Excel sheet to build the table and chart.

**Figure 8:**
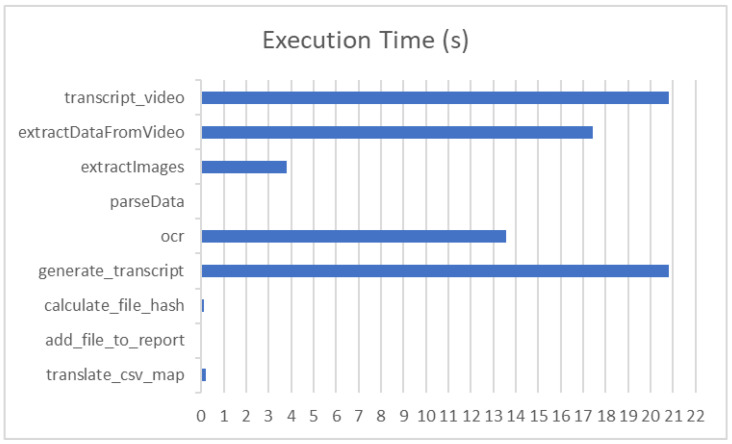
Execution time in seconds showing that the video-dependent functions consume much time.

Table 2 and Figure 8 show the execution time in seconds for the main functions that are responsible for processing each selected evidence video.

The table shows that the functions that depend on the video as input consume much computational time, e.g., OCR, transcription of the video and generation of transcripts. The other functions that are used for generating the digital forensic report are light and produce results in a short time.

**Figure 9:**
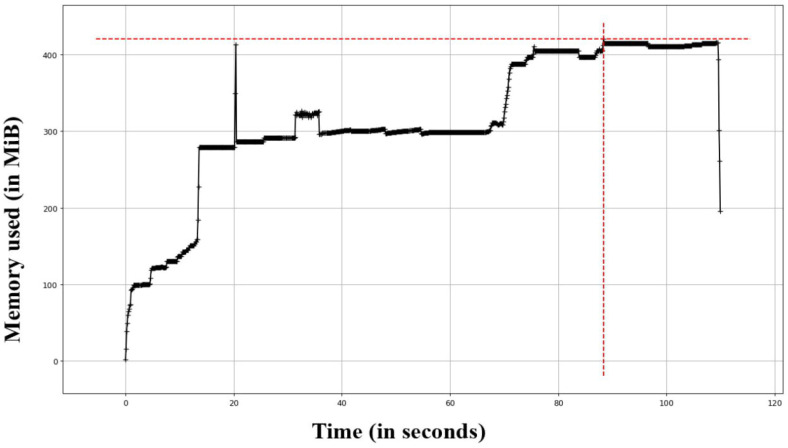
Memory usage in MiB, showing that more memory is needed when the length of the recording increases.

In order to perform memory usage analysis, memory_profiler is used to collect memory usage during the execution of the profiled functions. The function also uses matplotlib Python package to interpret the collected data and draw the memory usage chart, as shown in Figure 9. The tool memory_profiler represents memory usage in MiB (mebibytes) rather than MB (megabytes). A mebibyte is equal to 1,048,576 bytes, and a megabyte is equal to 1,000,000 bytes.

Figure 9 shows that more memory is required as the recording time increases. So the length of the recording also impacts the amount of memory used by the dashcam. Some dashcams are designed to record continuously, while others may only record when motion is detected or when an impact is detected. The longer the dashcam records, the more memory is used. There are also other factors affecting the memory usage. Dashcams typically record video in one of the following resolutions: 1080p, 720p or 480p. The higher the resolution, the more memory is used to store the video footage. Similarly, a higher frame rate, such as 30 frames per second (fps) or 60 fps, requires more memory than a lower frame rate.

### 5.4. Generating Digital Forensic Reports

After intensive testing of the developed dashcam forensics framework, the framework is found capable of extracting dashcam-specific artifacts. The framework is also capable of detecting collision and lane departure warnings that happened during the video recording in addition to transcribing the conversation recorded between the driver and passenger(s), if any. The detected lanes and approaching vehicles are drawn on the dashcam screen in real-time and are not recorded as part of the video. So collision warnings and lane departure warnings are converted into texts and stored in the database along with the GPS coordinates, time and speed. This enables investigators to track the vehicle while driving.

**Figure 10:**
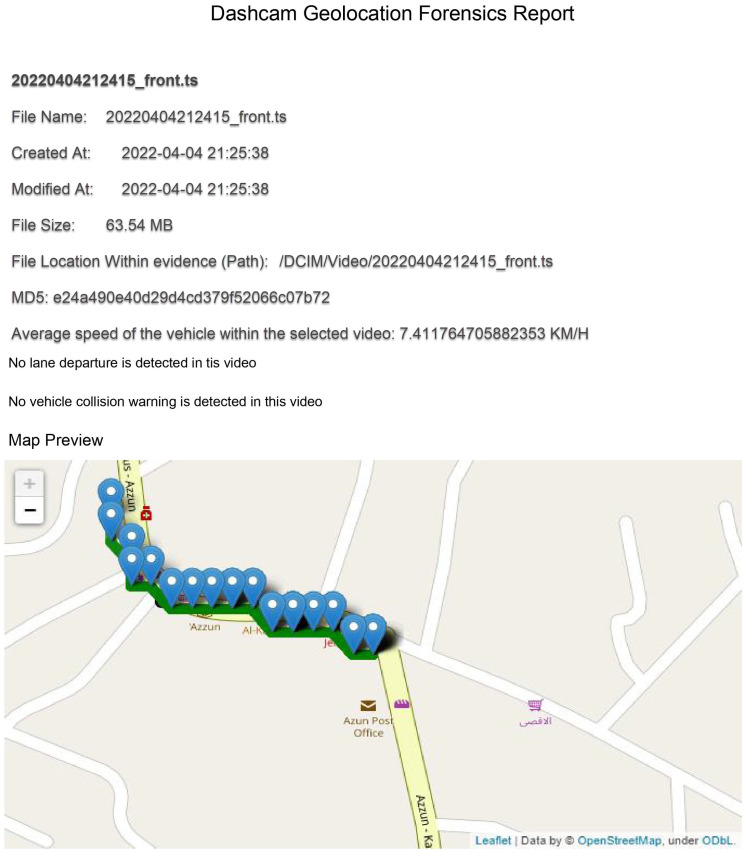
Interactive map of the selected video coordinates.

Additionally, the results also show that the framework is capable of processing given video evidence and reflecting it as GPS coordinates on an interactive map showing the trajectory where the video was recorded, the speed of the vehicle at the time of video recording and the date and time when every second of the video was recorded in addition to a preview snapshot for each second recorded in the video. We also found that the framework is capable of including all of the extracted artifacts from a given video into the digital forensic report, as shown in Figure 10.

Comparing the proposed framework with commercial tools, such as Belkasoft, this tool can handle the provided evidence image as a storage media that contains media files but does not support the flexibility of digging deeper inside each media file and extracting artifacts based on the way a certain media file is generated. In fact, the Belkasoft tool requires additional software for obtaining the details of the selected file, such as the MD5 hash. But when it comes to the content of the file itself, Belkasoft provides the ability to preview the file through performing a visual review by the investigator. Meanwhile, the proposed framework supports automatic review of files by extracting the vehicle speed, GPS coordinates and a transcription of the video file.

## 6. Conclusions

This paper has proposed a digital forensics framework for analyzing dashcam videos, which are considered evidence about vehicle incidents. The framework enables investigators to extract evidential metadata from dashcam SD cards and track the evidence over the entire trip. As evidence in the video can be in text and sound formats, we tested three OCR methods and found that Tesseract is the most accurate. Therefore, Tesseract can be used to extract time, date, speed, GPS coordinates and other necessary texts from the dash video. We also tested two speech-to-text conversion methods for extracting vocal warnings, such as for lane departures and collisions. We found that the Google speech-to-text conversion API is more accurate than Mozilla’s DeepSpeech. However, DeepSpeech is preferred, as its error rate is not high and it works offline, avoiding the risk of evidence modification on the network. By this framework, investigators obtain a method that associates temporal and geospatial artifacts from a specific dashcam onto a map, which supports evidence tracking. The framework allows investigators to print a report that contains the entire evidence story. The framework ensures evidence authenticity, as it is processed in one place instead of on several software tools.

The framework results may contain data that are not related to the incident under investigation, such as license plates of other vehicles or house numbers. This can be considered a privacy violation, but digital forensic investigators must adhere to ethical standards and legal requirements, such as obtaining search warrants before accessing personal data. These safeguards help to ensure that digital forensic investigations are conducted in a manner that respects the privacy of individuals and complies with legal and ethical guidelines [65]. Thus, future work is needed to preserve the privacy of all items in the dashcam videos. Also, dashcams require a well-lit environment to record high-quality video, which means that the accuracy of the recording is reduced at night. Also, when the video is recorded while vehicle windows are open, the quality of the recorded speech is affected and the accuracy of the speech-to-text conversion is reduced. Therefore, future work is also needed to process video and speech accompanied by high noise. This research agrees with [54], which shows that OCR techniques can become more robust if they are integrated with deep learning to achieve high accuracy in different weather conditions.

## Figures and Tables

**Table 1:** Comparison of speech-to-text conversion methods for dashcam videos.

Video Length in Minutes	Google API	DeepSpeech
10	4.2	6.5
15	4.7	7.9
18	5.1	8.7
24	5.3	10.4
33	5.8	11.5
47	4.9	10.7

**Table 2:** Execution time in seconds and percentage of each function.

Function Name	Execution Time (s)	Percentage
translate_csv_map	0.2066	0.269%
add_file_to_report	0.0054	0.007%
calculate_file_hash	0.1118	0.146%
generate_transcript	20.8220	27.101%
ocr	13.5920	17.691%
parseData	0.00163	0.002%
extractImages	3.8242	4.977%
extractDataFromVideo	17.4466	22.708%
transcript_video	20.8207	27.099%
Total	76.8311	100.00%

## Data Availability

Not applicable.
